# Exploring optimization strategies for improving explicit water models: Rigid n-point model and polarizable model based on Drude oscillator

**DOI:** 10.1371/journal.pone.0224991

**Published:** 2019-11-14

**Authors:** Yeyue Xiong, Alexey V. Onufriev

**Affiliations:** 1 Department of Biomedical Engineering and Mechanics, Virginia Tech, Blacksburg, VA, United States of America; 2 Department of Physics, Virginia Tech, Blacksburg, VA, United States of America; 3 Department of Computer Science, Virginia Tech, Blacksburg, VA, United States of America; 4 Center for Soft Matter and Biological Physics, Virginia Tech, Blacksburg, VA, United States of America; Universidade Nova de Lisboa Instituto de Tecnologia Quimica e Biologica, PORTUGAL

## Abstract

Rigid *n*-point water models are widely used in atomistic simulations, but have known accuracy drawbacks. Increasing the number of point charges, as well as adding electronic polarizability, are two common strategies for accuracy improvements. Both strategies come at considerable computational cost, which weighs heavily against modest possible accuracy improvements in practical simulations. In an effort to provide guidance for model development, here we have explored the limiting accuracy of “electrostatically globally optimal” *n*-point water models in terms of their ability to reproduce properties of water dimer—a mimic of the condensed state of water. For a given *n*, each model is built upon a set of reference multipole moments (*e.g*. *ab* *initio*) and then optimized to reproduce water dimer total dipole moment. The models are then evaluated with respect to the accuracy of reproducing the geometry of the water dimer. We find that global optimization of the charge distribution alone can deliver high accuracy of the water model: for *n* = 4 or *n* = 5, the geometry of the resulting water dimer can be almost within 5^0^ of the *ab initio* reference, which is half that of the experimental error margin. Thus, global optimization of the charge distribution of classical *n*-point water models can lead to high accuracy models. We also find that while the accuracy improvement in going from *n* = 3 to *n* = 4 is substantial, the additional accuracy increase in going from *n* = 4 to *n* = 5 is marginal. Next, we have explored accuracy limitations of the standard practice of adding electronic polarizability (via a Drude particle) to a “rigid base”—pre-optimization rigid *n*-point water model. The resulting model (*n* = 3) shows a relatively small improvement in accuracy, suggesting that the strategy of merely adding the polarizability to an inferior accuracy water model used as the base cannot fix the defects of the latter. An alternative strategy in which the parameters of the rigid base model are globally optimized along with the polarizability parameter is much more promising: the resulting 3-point polarizable model out-performs even the 5-point optimal rigid model by a large margin. We suggest that future development efforts consider 3- and 4-point polarizable models where global optimization of the “rigid base” is coupled to optimization of the polarizability to deliver globally optimal solutions.

## Introduction

**Fig 1:**
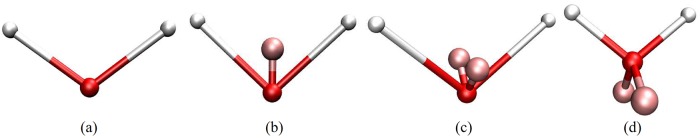
Rigid n-point water model geometries for n = 3, n = 4 and n = 5. Hydrogen, Oxygen and the extra interaction point with a point charge but no mass are represented as white, red and pink spheres respectively. 4, and 5-point water models have zero charge on their oxygen, the negative charge is placed on the extra point(s). (a) 3-point water model. (b) 4-point water model. (c) and (d) Different possible non-planar configurations of 5-point water models [18].

Water molecule has a deceptively simple structure H_2_O, yet many anomalies of liquid water are still hard to explain despite the countless studies [1–5]—not surprisingly, water in its liquid state is notoriously difficult to model. Many complex biomolecules (DNA, RNA, proteins, etc.), vital for a living cell’s function, cannot be studied alone without considering their environment—water as the solvent [6]. To study those large biomolecules, atomistic simulations have been widely used, and numerous different water models [7] have been developed to reproduce water properties, including the class of water models most widely used today—simple, rigid, fixed-charge explicit models such as TIP3P [8], TIP4P [9, 10], TIP5P [11] and SPC/E [12]. According to the convention, these classical water models are distinguished by the number of “points”—interaction sites—in them: 3-point models (Fig 1a, with 3 point charges) such as TIP3P are the most common due to what is perceived by many as an acceptable balance between accuracy and computational cost (compared with 4 or 5-point models). For water models with more points, 4-point models (Fig 1b, with 3 point charges and neutral oxygen) such as TIP4P, and especially 5-point models (Fig 1c and 1d, with 4 point charges and neutral oxygen) models such as TIP5P (Fig 1d), the cost considerations become significant, even though these models tend to reproduce water properties better than their 3-point counterparts. However, despite decades of effort by many groups, none of the existing simple water models is perfect. [7, 13–17]

It is known that liquid water properties are determined by a complex network of hydrogen bonds. In these classical water models, hydrogen bonds are mimicked mostly by the electrostatics, complemented by a Lennard-Jones (LJ) potential. The latter—LJ potential—is generally represented by a single site centered on the oxygen, and its corresponding interaction is isotropic and featureless, in contrast to hydrogen bonding, which is directional. Thus, accurately representing electrostatic interactions is paramount for a classical water model to mimic hydrogen bond interactions and reproduce liquid water properties [19]. Therefore, our first question is how accurately can we describe the reference electrostatics within the unavoidable limitations of *n*-point models, and how far it can get us in terms of water model accuracy, by optimizing the electrostatics alone. This is a non-trivial question since, by construction, these simple *n*-point rigid models miss many important physics, such as molecular flexibility, electronic polarizability, and charge transfer.

We note that a good water model intended for biomolecular simulations must reproduce experimental properties of water with high accuracy. There are at least two reasons why existing water models are far from perfect in this respect. One is the limitations of the optimization strategies used in constructing water models. The other reason is on a more fundamental level—the limited physics built into these simple models. As an example of the first kind of limitation, current widely used rigid 3-point models (TIP3P, SPC, SPC/E, etc.) are based on an assumption that the experimental water molecule geometry is somehow optimal, or near optimal, for a classical water models—consequently, these models place the point charges on, or near, the centers of hydrogen and oxygen atoms. While sophistication of the optimization techniques employed to find the optimum has grown tremendously [20], from essentially “guess-and-test” to the complex, state-of-the-art force balance optimizations [21], one crucial aspect of the over-all procedure has not changed until recently: the search for best fit model is performed in the vicinity of the “canonical” water geometry, thus returning a *local optimum* in the parameter space. It was recently demonstrated [19] that abandoning the restrictions (except fundamental *C*_2*v*_ symmetry) on water model geometry, and performing an exhaustive search for a truly *global* optimum in the parameter space can result in significant accuracy improvements. The resulting 4-point rigid model—OPC [19] was built without the geometry restriction on point charge placements. It was optimized globally to reproduce bulk water properties as best as possible, without any increase of the computational cost of employing the model in simulations, relative to common 4-point models such as TIP4P-Ew.

Apart from using global optimization, a natural strategy to improve the accuracy of a rigid *n*-point water model is to consider larger “*n*”. The key question with this approach is whether the inevitable and substantial increase in computational cost of employing “larger n” water models is justified by significantly better accuracy? If the accuracy gain is large enough, the cost increase may still be well worth it. Answering the question is important both for model developers and practitioners alike. Unfortunately, it is nearly impossible to compare existing *n*-point water models on the same footing to find out exactly how much gain an increase in *n* brings. For once, these models are optimized using different criteria, and against non-identical set of water properties. For example, TIP5P-Ew model [22] surpasses many of its 3- and 4- point predecessors in accuracy of describing water structure, but its accuracy of reproducing the self-diffusion is lower than that of TIP4P-Ew. In addition, optimization protocols vary widely, and, except for OPC-family models, there is no guarantee that the model corresponds to the global optimum in the optimization landscape. Thus, it is entirely possible that a global optimum for a smaller *n* may yield a more accurate model than a local one for a larger *n*.

Even if a hypothetically perfect fixed-charge rigid model reproduced a large subset properties of bulk liquid water exactly, the model would still be inherently incapable of responding to the change of polarity of its micro-environment, relevant to biomolecular simulations. Specifically, water is highly polarizable [23]: the experimentally observed change in the dipole moment of real water molecule upon transfer from gas to liquid phase is as large as 1 Debye, while for any rigid fixed-charge model that change is zero by construction. The polarity of micro-environment near a macromolecule can be quite different from that of bulk water; non-polarizable models cannot properly respond to different micro-environments during the course of a simulation [24], *e.g*. in cross-membrane transport. However, the vast majority of current water models lack polarizability, which must adversely affect the accuracy of biomolecular simulations. This example illustrates the second type of limitation on water model accuracy—the missing physics. To address the lack of electronic polarization, a number of polarizable water models have been developed, for example POL1 [25, 26], FF12POL [27], SWM model family [28] and AMOEBA model family [29–31], see recent reviews for a comprehensive account of the field [7, 32, 33]. There is little doubt that, in principle, availability of highly accurate and efficient polarizable models for routine practical simulations should improve the accuracy of biomolecular simulations significantly, by accounting for effects completely missed by rigid fixed-charge models. However, because of the unavoidable higher complexity of polarizable models relative to the fixed-charge ones, the balance towards wider adoption can only be tipped by distinctly higher accuracy, which does not seem to be the case yet [27, 34]. Since current polarizable water models typically use existing fixed-charge models as their “base”, they likely inherit at least some of the existing flaws of the fixed-charge models, unrelated to their lack of polarizability. That is they are likely represented by local optima in the complex optimization landscape. Indeed, despite undeniably better physical foundation, accuracy of a sophisticated polarizable water model can be lower than that of a globally optimal non-polarizable rigid model [19]. This observation begs the question: how much accuracy gain can the inclusion of polarizability bring, if the above optimization-related limitations were removed, and a truly globally optimal polarizable model was constructed? In other words, is global optimization a potentially useful strategy for polarizable models? For reasons discussed above in the context of comparing non-polarizable models, comparing existing *n*-point non-polarizable with polarizable models (even with respect to liquid water properties alone) on the same footing is difficult. Moreover, to the best of our knowledge, a truly globally optimal polarizable model has not be constructed yet.

The main motivation of this work is to address the questions outlines above in a tightly controlled computational experiment. To this end, here we build and evaluate several test water models of two types—rigid *n*-point globally optimal models (*n* = 3, 4, 5) and also polarizable models based on these rigid models—that illustrate the two limitations discussed above. We also construct a truly globally optimal polarizable test model. All models are optimized using the same type of protocols, and to the same accuracy level in reproducing the training set. The models are evaluated by examining their ability to reproduce water dimer, which can be considered as the simplest possible mimic of water in condensed state.

## Methods

As mentioned above in the Introduction, liquid water properties are determined by a complex network of hydrogen bonds, which is the most challenging part a water model needs to mimic in order to simulate real water. Water dimer, involving only two water molecules and their interactions, is a good minimal starting point to reveal the secrets of hydrogen bonding, and has been studied over half a century [35, 36]. Due to its significance to hydrogen bonding studies, and being the simplest possible mimic of the liquid phase water, the water dimer structure is utilized here to evaluate the quality of the water models.

### Overall approach

**Fig 2:**
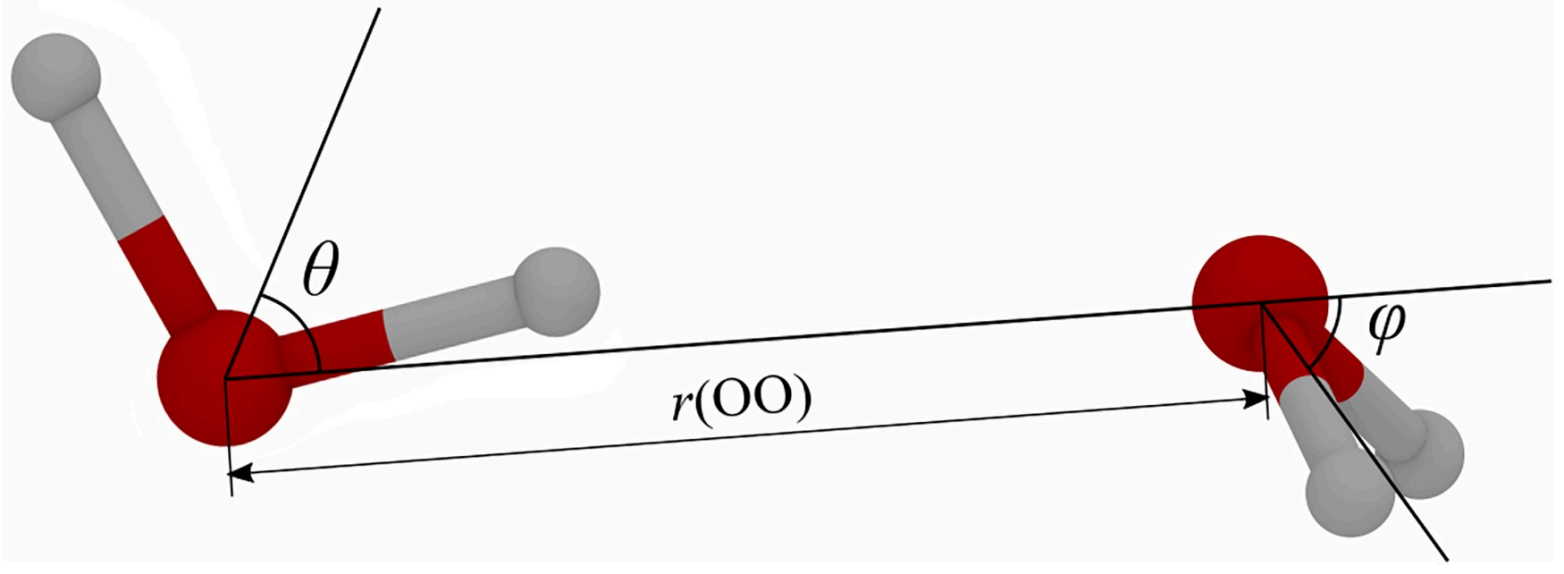
Schematic of a water dimer. *r*(OO) is the distance between the two oxygens; *θ* and *φ* are the angles between water molecule’s *C*_2_ axis and the O-O line for the two molecules respectively [18].

The overall approach is as follows. Individual water model’s geometry and values of point charges are optimized to match as closely as possible a set of reference multipole moments (Table 1, below). The matching is done in the following precise sense. Through optimal point charge approximation (OPCA, a method to approximate electrostatic charge distributions with a small number of point charges to optimally represent the original charge distribution) [37], each water model has its quadrupole and octupole moments fitted to the reference, and the monomer dipole moment is optimized so that when the two water models form a dimer, the dimer total dipole has a smaller than 0.1% error relative to *ab* *initio* calculation—2.68D [38]. Then the water model’s accuracy is evaluated by comparing its dimer’s geometry (Fig 2) with the one obtained from an *ab* *initio* calculation [39].

**Table 1:** Reference multipole moments of single water molecule.

data sets	*μ*/D	*Q*_*t*_/DÅ	*Q*_0_/DÅ	Ω_*t*_/DÅ^2^	Ω_0_/DÅ^2^
gas phase experimental [37]	1.86	2.57	0.11	—	—
gas phase QM calculation [37]	1.81	2.49	0.08	1.93	-1.35
liquid phase MP2/4MM calculation [47]	2.49	2.93	0.13	2.09	-1.73

As discussed in the introduction, a water model’s accuracy of simulating liquid water is largely determined by its electrostatic properties, small variations in these properties can lead to large differences in simulation outcomes [40]. Also, the “electrostatic” parameters are where different types of water models differ the most from each other, while the Van der Waals potential is typically simulated in the same manner: by a Lennard-Jones (LJ) site on oxygen. All of model types studied here follow that approach. In reality, optimization of the LJ parameters can be largely decoupled from optimization of the “electrostatic” parameters such as partial charges and their distribution [19]. An accepted approach to construct realistic water models involves optimizing the LJ parameters against reference O-O RDF (radial distribution function), to obtain a close match [19]; we follow essentially the same strategy here, except that we use *ab-initio* reference oxygen-oxygen distance (*r*(*OO*) = 2.91*Å* [38, 39]) as the target reference for optimization of LJ parameters. With the *r*(*OO*) distance used in LJ optimization, the remaining parameters of the dimer geometry—the two angles (*θ* and *φ* in Fig 2)—are left as convenient metrics to evaluate the model accuracy, which is mainly determined by the electrostatics.

From the perspective of electrostatics, a model with larger *n* (number of interaction points) is expected to better reproduce given multipole moments, and thus improve the accuracy of the model [41–44].

For a set of *N* point charges, in this case—the water model, the Coulomb potential can be written as:

$$\begin{array}{c} \varphi \left(R\right)=\frac{1}{4\pi \epsilon_{0}}\sum_{n=1}^{N}\frac{q_{n}}{∥R-r_{n}∥} \end{array}$$ (1)

In a Cartesian system, this equation becomes:

$$\begin{array}{cc} \varphi (R)= & \frac{1}{4\pi \epsilon_{0}}(\frac{1}{R}q+\frac{1}{R^{2}}\sum_{i=x,y,z}{\hat{R}}_{i}\mu_{i}+\\ & \;\frac{1}{R^{3}}\sum_{i,j=x,y,z}{\hat{R}}_{i}{\hat{R}}_{j}Q_{ij}+\\ & \;\frac{1}{R^{4}}\sum_{i,j,k=x,y,z}{\hat{R}}_{i}{\hat{R}}_{j}{\hat{R}}_{k}O_{ijk}+\ldots ) \end{array}$$ (2)

$$\begin{array}{c} q=\sum_{n=1}^{N}q_{n} \end{array}$$ (3)

$$\begin{array}{c} \mu_{i}=\sum_{n=1}^{N}q_{n}r_{n,i} \end{array}$$ (4)

$$\begin{array}{c} Q_{ij}=\frac{1}{2}\sum_{n=1}^{N}q_{n}\left(3r_{n,i}r_{n,j}-{\left(r_{n}\right)}^{2}\delta_{ij}\right) \end{array}$$ (5)

$$\begin{array}{cc} O_{ijk}= & \frac{1}{6}\sum_{n=1}^{N}q_{n}[15r_{n,i}r_{n,j}r_{n,k}-\\ & \;3{\left(r_{n}\right)}^{2}\left(r_{n,i}\delta_{jk}+r_{n,j}\delta_{ik}+r_{n,k}\delta_{ij}\right)] \end{array}$$ (6)

Because of the *C*_2_ symmetry of a water molecule, the quadrupole moments and the z-plane elements of octupole moments can be written as:

$$\begin{array}{cc} Q= & \left[\begin{array}{ccc} -Q_{t}-Q_{0}/2 & 0 & 0\\ 0 & Q_{t}-Q_{0}/2 & 0\\ 0 & 0 & Q_{0} \end{array}\right] \end{array}$$ (7)

$$\begin{array}{cc} O_{ijz}= & [\begin{array}{ccc} -\Omega_{t}-\Omega_{0}/2 & 0 & 0\\ 0 & \Omega_{t}-\Omega_{0}/2 & 0\\ 0 & 0 & \Omega_{0} \end{array}]\text{where}\;i,j\;\text{range}\;\text{over}\;x,y\;\text{and}\;z \end{array}$$ (8)

With the equations all set up, we can then rearrange them to the form where water model parameters (coordinates and charge) are explicitly expressed with multipole moments [19].

For a 3 or 4-point water model, the rearranged equations become:

$$\begin{array}{cc} & z_{1,2}=\frac{2Q_{t}+3Q_{0}}{6\mu }\mp \frac{\mu }{4q} \end{array}$$ (9)

$$\begin{array}{cc} & y=\sqrt{\frac{2Q_{t}}{3q}} \end{array}$$ (10)

$$\begin{array}{cc} & q=-3\frac{\sqrt{\mu^{4}\left(256Q_{t}^{2}+\xi \right)}+16Q_{t}\mu^{2}}{2\xi } \end{array}$$ (11)

$$\begin{array}{cc} \text{where}\;\xi = & 52Q_{t}^{2}+60Q_{T}Q_{0}-\\ & 9[3Q_{0}^{2}+8\left(\Omega_{t}-\Omega_{0}/2\right)\mu ] \end{array}$$ (12)

**Fig 3:**
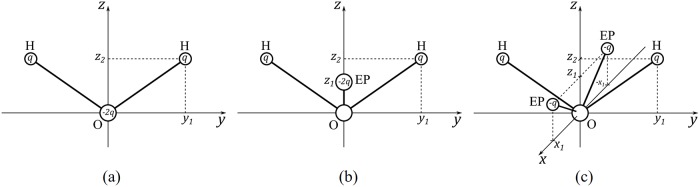
Schematics of 3, 4 and 5-point rigid water models. Each circle represents an interaction point (H, O atom, or extra point–EP). Thick lines are chemical bonds. The origin point is on the Oxygen; *C*_2_ symmetry axis of water is set as the z-axis; y-axis is in the H-O-H plane and passes through O; x-direction is only accounted for in a 5-point model, and the 2 extra interaction points (EP) are placed on the *xOz* plane. Electric charge on H is *q*, charge −2*q* is placed on O for 3-point model, on EP for 4-point model, and divided evenly on the two EPs for 5-point model. (a) 3-point water model. (b) 4-point water model. (c) 5-point water model.

A 4-point water model is shown in Fig 3b. With the *C*_2_ symmetry, only 4 parameters are needed to construct the 4-point water model—*y*_1_, *z*_1_, *z*_2_ and electric charge *q*. Both 3 and 4-point models have 3 point charges, so the calculation process are the same (Eqs 9–12) except for 3-point models the condition *z*_1_ = 0 needs to be imposed (Fig 3a).

For a 5-point model, there are 5 parameters to be determined (*q*, *x*_1_, *y*_1_, *z*_1_, *z*_2_, see Fig 3c).

From Eqs (3)–(6), we have:

$$\begin{array}{cc} \mu & =2q(z_{2}-z_{1}) \end{array}$$ (13)

$$\begin{array}{cc} Q_{t} & =\frac{3}{2}q\left(x_{1}^{2}+y_{1}^{2}\right) \end{array}$$ (14)

$$\begin{array}{cc} Q_{0} & =q(x_{1}^{2}-y_{1}^{2}-2z_{1}^{2}+2z_{2}^{2}) \end{array}$$ (15)

$$\begin{array}{cc} \Omega_{t} & =\frac{5}{2}q\left(x_{1}^{2}z_{1}+y_{1}^{2}z_{2}\right) \end{array}$$ (16)

$$\begin{array}{cc} \Omega_{0} & =q(3x_{1}^{2}z_{1}-3y_{1}^{2}z_{2}-2z_{1}^{3}+2z_{2}^{3}) \end{array}$$ (17)

With Eqs (13)–(16), a series of expression (*x*_1_, *y*_1_, *z*_1_, *z*_2_) = *f*(*q*, *μ*, *Q*_*t*_, *Q*_0_, Ω_*t*_) can be derived:

$$\begin{array}{cc} x_{1} & =\sqrt{\frac{-36\Omega_{t}\mu q+30Q_{0}Q_{t}q+20Q_{t}^{2}q+15Q_{t}\mu^{2}}{q(60Q_{t}q+45\mu^{2})}} \end{array}$$ (18)

$$\begin{array}{cc} y_{1} & =\sqrt{\frac{36\Omega_{t}\mu q-30Q_{0}Q_{t}q+20Q_{t}^{2}q+15Q_{t}\mu^{2}}{q(60Q_{t}q+45\mu^{2})}} \end{array}$$ (19)

$$\begin{array}{cc} z_{1} & =\frac{48\Omega_{t}q^{2}+30Q_{0}\mu q-20Q_{t}\mu q-15\mu^{3}}{q(80Q_{t}q+60\mu^{2})} \end{array}$$ (20)

$$\begin{array}{cc} z_{2} & =\frac{48\Omega_{t}q^{2}+30Q_{0}\mu q+20Q_{t}\mu q+15\mu^{3}}{q(80Q_{t}q+60\mu^{2})} \end{array}$$ (21)

After choosing a set of multipole data (*μ*, *Q*_*t*_, *Q*_0_, Ω_*t*_, Ω_0_), insert *μ*, *Q*_*t*_, *Q*_0_, Ω_*t*_ values into Eqs (18)–(21) so that we can then express the coordinates (*x*, *y*, *z*_1_, *z*_2_) with one argument—*q* the charge value. And now, what is left is a very simple optimization problem: finding the optimal *q* that makes the Ω_0_ error of the model as small as possible with exhaustive search. Then with the optimal *q* and its corresponding coordinates (*x*, *y*, *z*_1_, *z*_2_), a 5-point water model is then constructed and ready for evaluation.

Evaluation of the built water models starts with the energy minimization calculation of two identical water molecules that form a water dimer, using AMBER 2019 [45]. Steepest descent, conjugate gradient method (for 3, 4-point rigid models) and Limited-memory Broyden-Fletcher-Goldfarb-Shanno (LBFGS) quasi-Newton algorithm (for 5-point rigid models and 3-point Drude models) are used in this minimization calculation. With the energy minimization process, the LJ parameters on the oxygen of each model are determined so that the minimized water dimer has an oxygen-oxygen distance matching the reference (2.91Å). With the oxygen-oxygen distance controlled, the dimer system has a very limited number of variables (rotation angles), therefore finding the global minimum is straightforward. To assert it, we ran multiple minimizations with random starting coordinates, and found that all the minimized states were the same. Thus, after minimization, the dimer system of the constructed water model reaches its global lowest energy state. The total dipole moment of the minimized water dimer is then calculated and compared with the ab initio result(2.68D [38]). If the relative error of the total dipole moment is greater than 0.1%, the monomer dipole moment will be changed, we then redo the model building and the dimer minimization. This optimization process is continued until a monomer dipole value is found when the total dipole moment of the minimized dimer has a relative error within 0.1%.

As a metric to evaluate the geometry of water dimer, two parameters are used—*θ* and *φ*. *θ* and *φ* are the angles between water molecule’s *C*_2_ axis and the O-O line for the two molecules respectively. *θ* is of the water molecule coplanar with O-O, *φ* is of the other one non-coplanar with O-O (Fig 2). We use an *ab* *initio* calculation of a water dimer as the reference geometry, where *θ*_0_ = 57.9°, *φ*_0_ = 55.6° and *r*(OO) = 2.91*Å*. [38, 39] This *ab* *initio* calculation is done by Klopper et al. in 2000 [39], optimized at the level of CCSD(T) theory with IO249 basis. It is seen as a benchmark result of theoretical studies on water dimer. [36]

### Reference multipole moment sets

To ascertain robustness of our methodological conclusions, we use three different sets multipole moments as references to which we fit our water models (Table 1). The monomer dipole, quadrupole and octupole moments of gas phase QM calculation (CCSD with aug-cc-pCVTZ basis) are from Anandakrishnan et al [37]. The gas phase experimental data are from Clough et al [46]. When used to construct water models, the octupole moments from the gas QM set are used in the gas phase experimental set (whose octupole moments are not available). The third set of reference multipole moments is of liquid phase water, a QM calculation done by Niu et al [47].

Each set of reference multipole moments is used to construct a set of optimal water models comprising of 3-point, 4-point and 5-point rigid models. These three sets of water models are called “gas exp.”, “gas QM” and “liquid MP2/4MM” respectively, based on the dataset used for model construction. The “gas exp” reference multipole moments are also used to construct polarizable models in this study.

### n-point rigid models

**Fig 4:**
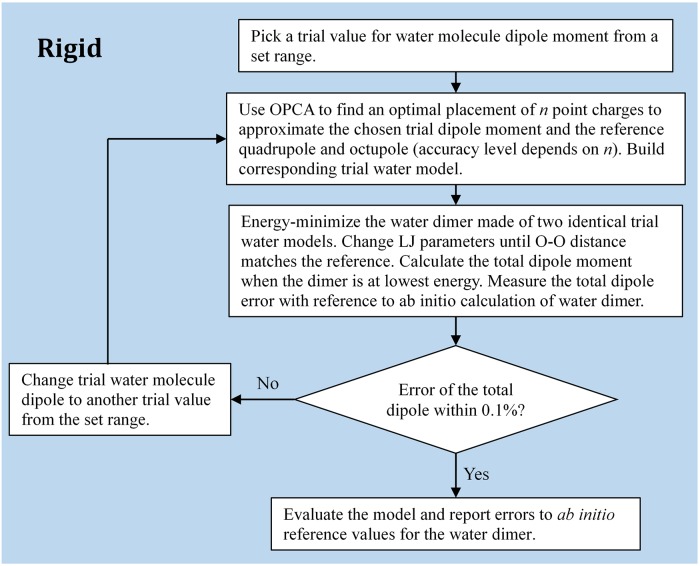
Flowchart: The process of building a rigid *n*-point water model.

Optimized against each of the three multipole moment data sets (“gas exp.”, “gas QM” and “liquid MP2/4MM”), three rigid models were constructed with 3, 4 and 5 interaction points respectively, following the process shown in Fig 4. Parameters of these rigid models are listed in Table 2.

**Table 2:** Parameters of 3, 4 and 5-point optimal water models, and their geometry (Fig 1). For comparison, parameters of several existing models are shown in the bottom rows.

Name or reference	*n*	Type (Fig 1)	*q*[e]	*z*_1_[Å]	*z*_2_/[Å]	*y*_1_[Å]	*x*_1_[Å]	*μ*[D]	*σ*_*LJ*_[Å]	*ϵ*_*LJ*_[kJ/mol]
Gas exp.	3	a	0.2202	—	0.9284	1.2727	—	1.964	3.3320	0.7113
	4	b	1.9094	0.3351	0.4601	0.4322	—	2.293	3.2999	0.7196
	5	c	0.8633	0.1862	0.4723	0.6179	0.1773	2.373	3.2945	0.7740
Gas QM	3	a	0.2217	—	0.9039	1.2486	—	1.925	3.2999	0.7573
	4	b	1.4346	0.3073	0.4698	0.4908	—	2.239	3.2910	0.7071
	5	c	0.9194	0.2184	0.4769	0.5989	0.1314	2.283	3.2749	0.7782
Liquid MP2/4MM	3	a	0.2261	—	0.9795	1.3413	—	2.127	3.3854	0.7448
	4	b	2.7937	0.3679	0.4615	0.3815	—	2.512	3.3516	0.7364
	5	c	0.5833	0.0234	0.5076	0.7632	0.3386	2.713	3.3801	0.7699
TIP3P	3	a	0.4170	—	0.5859	0.7570	—	2.35	3.1506	0.6364
OPC3	3	a	0.4476	—	0.5652	0.7992	—	2.43	3.1743	0.6837
OPC	4	b	0.6791	0.1594	0.5395	0.6856	—	2.48	3.1666	0.8904

For each *n*-point rigid water model built, its monomer dipole moment *μ* value is varied for optimization. When the monomer dipole moment changes, the resulting water model parameters (coordinates and charges) change, as a result, the water dimer of this model will be different. An optimal monomer dipole moment is chosen when the total dipole of the corresponding dimer is within 0.1% relative error from the *ab* *initio* calculation [38].

### Polarizable water model with Drude oscillator

**Fig 5:**
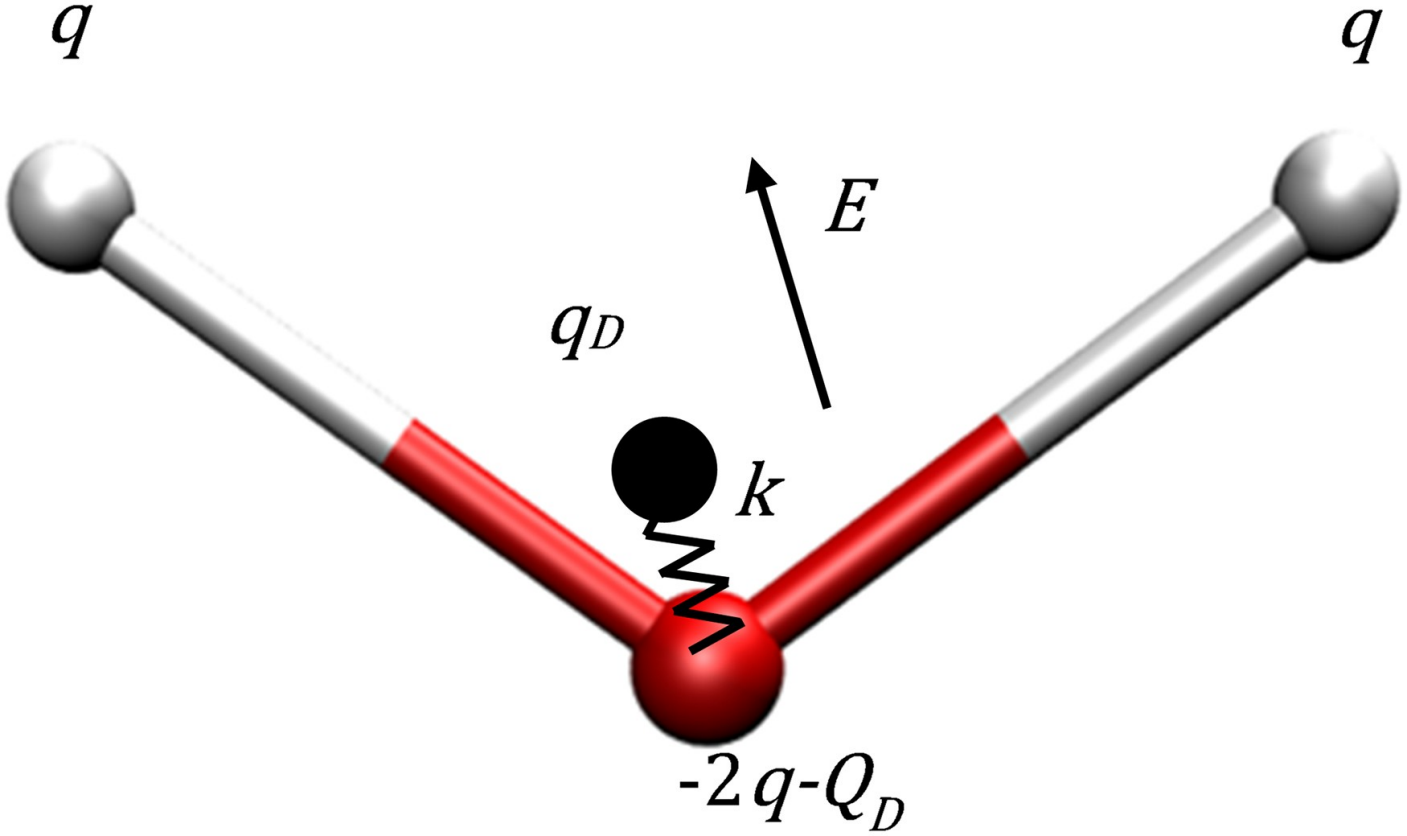
Schematic of a 3-point polarizable water model with Drude oscillator. This model is composed of three fixed point charges and a Drude oscillator particle attached to the Oxygen atom. Charge *q* is assigned to each white point as Hydrogen, charge (−2*q* − *Q*_*D*_) is assigned to the red point as Oxygen and the black point as the Drude particle has charge *Q*_*D*_ and zero mass. The black point is connected to the red point through a virtual bond with equilibrium length 0, force constant *k* and no directional restrictions. An external electric field *E* moves the Drude particle from its equilibrium position [18].

There are several different approaches available to add electronic polarizability to a “rigid base” water model, adding the Drude oscillator [48, 49] being one of the simplest methods. The Drude oscillator is easy to implement in most MD simulation packages. Critically, it only adds one more degree of freedom for optimization, which will not impair the search for the global optimum. Compared with other approaches, the Drude approach also has the benefit of high computational efficiency [32, 50–54]. Here the polarizable water model is constructed by adding a Drude particle to the oxygen in a 3-point rigid water model (Fig 5, the Drude particle has no interactions with the 2 hydrogens in the same molecule). It is referred to as a “3-point polarizable model”. The polarizability of this model is

$$\begin{array}{c} \alpha =Q_{D}^{2}/k \end{array}$$ (22)

where *k* ∼ 1000 kcal/mol/*Å*^2^ is the force constant of the virtual bond connecting the Drude particle and the Oxygen [55], *Q*_*D*_ is the charge of the Drude particle. In this study, we apply *k* = 1000 kcal/mol/Å^2^ and keep it constant. The charge on the Drude particle is set to

$$\begin{array}{c} Q_{D}=\sqrt{\alpha k}\times 18.2223\;\text{e} \end{array}$$ (23)

where the unit of *α* is *Å*^3^. The coordinates and charges of the fixed three points are calculated with the same OPCA method used in constructing 3-point rigid models.

Based on “gas exp.” data set (Table 1), we built two polarizable models: the “limited optimal” one with only polarizability optimized for fair comparison with rigid models; and the “globally optimal” one with both polarizability and gas phase monomer dipole moment optimized for practical evaluation.

#### Optimizing the 3-point polarizable water model

**Fig 6:**
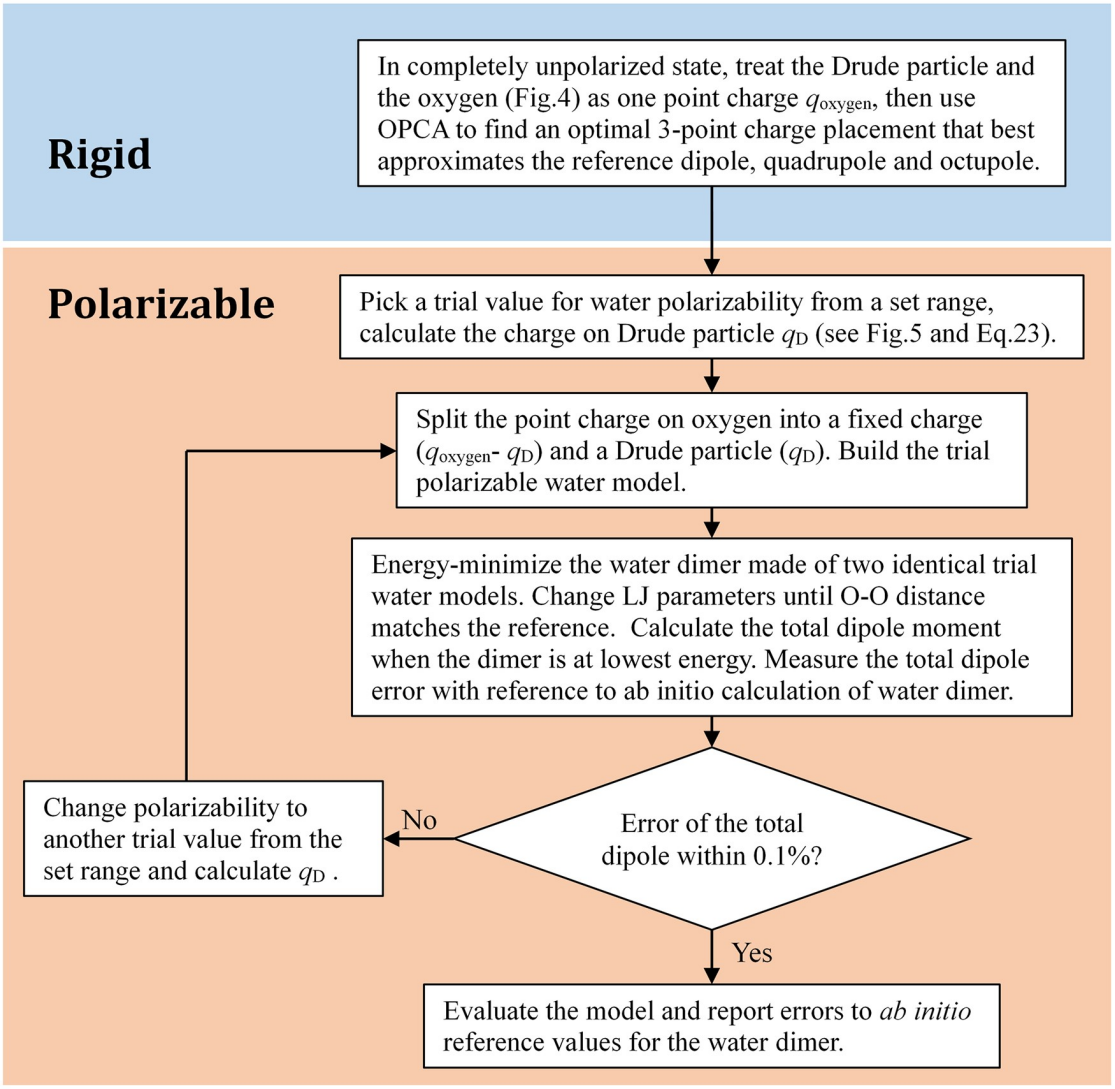
Flowchart: The process of building a polarizable Drude water model.

**Fig 7:**
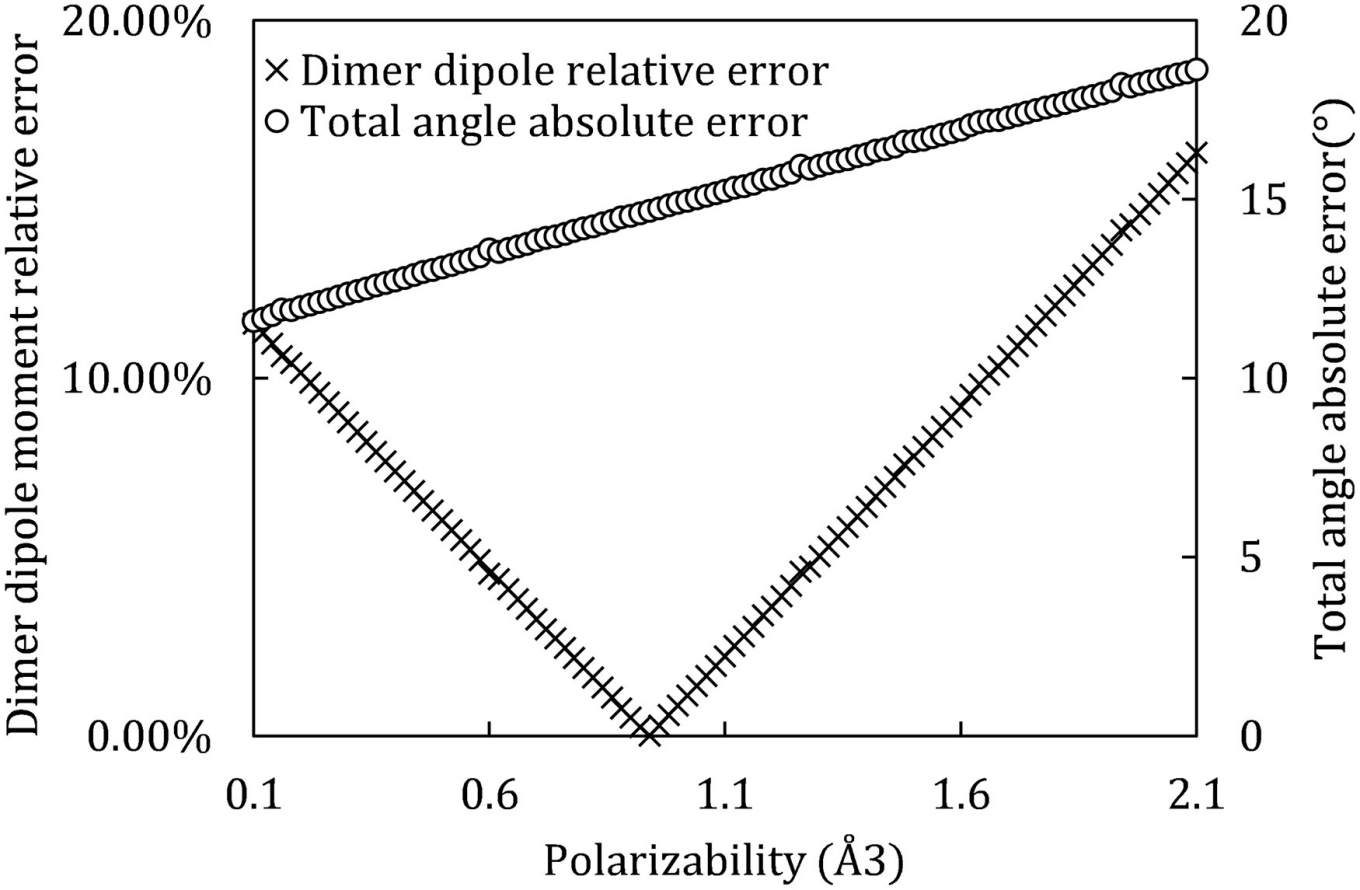
Dimer total dipole moment and total angle error of the limited optimal 3-point polarizable model. With the water monomer dipole fixed at 1.86D, we test different 3-point polarizable models by varying the polarizability from 0.10Å^3^ to 2.10Å^3^. The blue diamond symbols represent the total dipole errors and the orange triangle symbols show the total angle errors, where both are compared with the *ab* *initio* reference [38, 39].

For the limited optimal 3-point polarizable model, we fixed the monomer dipole moment to experimental value–1.86D (thus the rigid base is fixed), and optimized it by varying polarizability until its dimer dipole reproduces experimental result with in 0.1% error, the procedure is shown in Fig 6. The optimization and the resulting model are shown in Fig 7 and Table 3, respectively. The resulting polarizability of the limited optimal polarizable model is 0.94Å^3^, 35% lower than the gas phase experiment value–1.44Å^3^ [55].

**Table 3:** Parameters of the 3-point polarizable water models.

Optimization	*n*	*q*[*e*]	*Q*_*D*_[*e*]	*z*_2_[*Å*]	*y*_1_[*Å*]	$\bar{\alpha }\left[{Å}^{3}\right]$	$\bar{\mu }\left[\text{D}\right]$	*σ*_*LJ*_[Å]	*ϵ*_*LJ*_[kJ/mol]
*α*	3	0.1975	1.6825	0.9803	1.3439	0.94	1.86	3.3836	0.7113
*α* & *μ*	3	0.1700	2.6261	1.0565	1.4484	2.2900	1.7258	3.3786	1.0435

**Fig 8:**
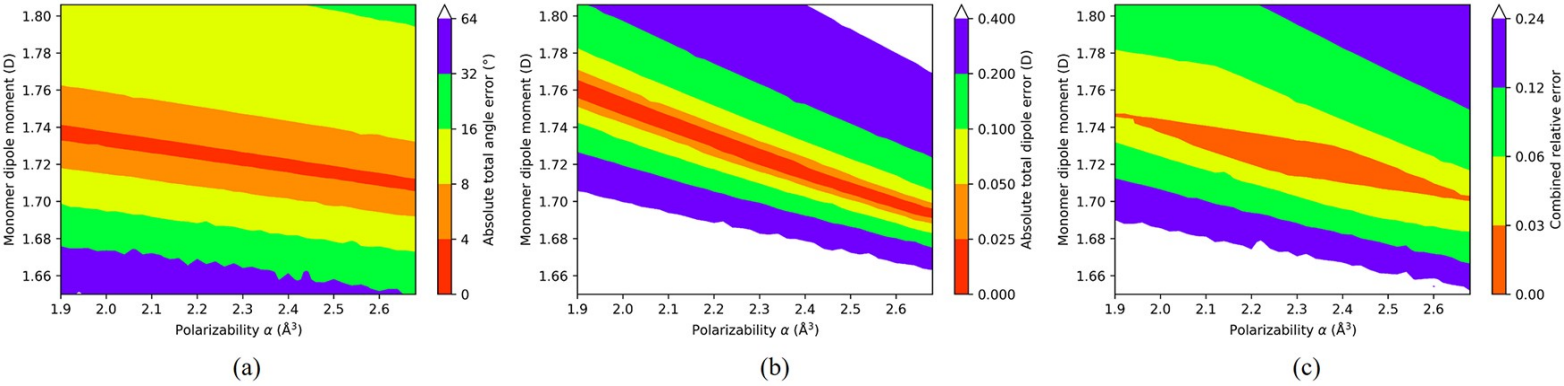
Globally optimal polarizable model error distribution. (a) The absolute error of the total angle (*θ* + *φ*) as a function of the corresponding model’s monomer dipole *μ* and polarizability *α* value. (b) The water dimer total dipole moment error with respect to the model’s *μ*, *α* combination. (c) The approximate range of the optimal *μ*, *α* combinations, where the “combined relative error” is defined as *Error*_*combined*_ = *Error*_*angle*_/180° + *Error*_*dipole*_/*p*_*dimer*_ with *p*_*dimer*_ = 2.68D [38]. The “combined relative error” was not used in the actual optimization process.

For the globally optimal 3-point polarizable model, we modify the model by changing two input parameters—monomer dipole moment *μ* and polarizability *α* (both in the vicinity of their gas phase experimental value, *μ*_*exp*_ = 1.86D and *α*_*exp*_ = 1.44Å^3^), to make the model’s dimer total dipole moment fall within 0.1% error from reference [39]. Error distributions of the intermediate models during this optimization process are shown in Fig 8, with respect to the two varying parameters.

The resulting globally optimal 3-point polarizable model is constructed with polarizability *α* = 2.2900Å^3^ and monomer dipole moment *μ* = 1.7258D (Table 3). This model has a total angle error of 3.19° when simulating a water dimer. The monomer dipole moment (1.7258D) of this polarizable model is relatively close to the gas phase experimental data (1.86D), while its polarizability (2.275Å^3^) is larger than the gas phase experimental value (1.44Å^3^).

## Results and discussion

For biomolecular simulations, the most important state of water is the liquid state at ambient temperature and pressure. However, the number of experimental parameters used to characterize liquid water is vast, and existing practical procedures used to build water models to match a subset of these parameters are highly diverse, non-trivial and expensive [7]. The process is still somewhat of an art. For a strict, limiting-case apples-to-apples comparison we want to present here, using liquid state properties is thus not ideal, if not completely impossible. We therefore resort to using a mimic of condensed state of water—water dimer. Water dimer is the smallest form of a water cluster. It has a hydrogen bond, which greatly contributes to numerous anomalies in water properties. As a starting point to study water properties, water dimer has been used extensively [35, 36, 56]. It has also been employed as a standard reference to test water models [8, 9, 11, 12, 19, 27–30].

Our over-all strategy is as follows (see Methods for details). For n-point rigid models, we build each model to match, as closely as possible, the reference quadrupole, octupole moments of water monomer (Table 1), and a monomer dipole moment optimized for water dimer. For polarizable models, its rigid “base” is built first to match the reference multipole moments including dipole, and then the polarizable model is built by adding a Drude oscillator with an optimal polarizability value—optimized for water dimer—to the rigid “base”. Parameters of the “base” are not re-optimized. The optimization of the last 3-point polarizable model is slightly different: it involves varying both the monomer dipole and the polarizability, such that both its “base” and Drude oscillator are optimized. Regardless of what parameters are optimized, the standard of the optimization is the same for all these models—the total dipole moment of the water model’s dimer matches the *ab* *initio* reference value for water dimer dipole [39] within a given tolerance.

We then test each model’s ability to reproduce the geometry of a water dimer formed by the two water model molecules. The reference dimer geometry is from the benchmark *ab* *initio* calculation by Klopper et al. [38, 39] For reasons detailed in “Methods”, we keep the O-O distance in the dimer fixed to its reference value, thereby excluding oxygen LJ parameters from the consideration.

### Rigid *n*-point water models

First we explore accuracy of rigid non-polarizable water models with 3, 4 and 5 point charges. The model parameters are given in Table 2. Three sets of *n*-point (*n* = 3, 4, 5) rigid water models are included. These three sets of water models are constructed based on 3 different sets of multipole moments data, see “Methods”.

**Fig 9:**
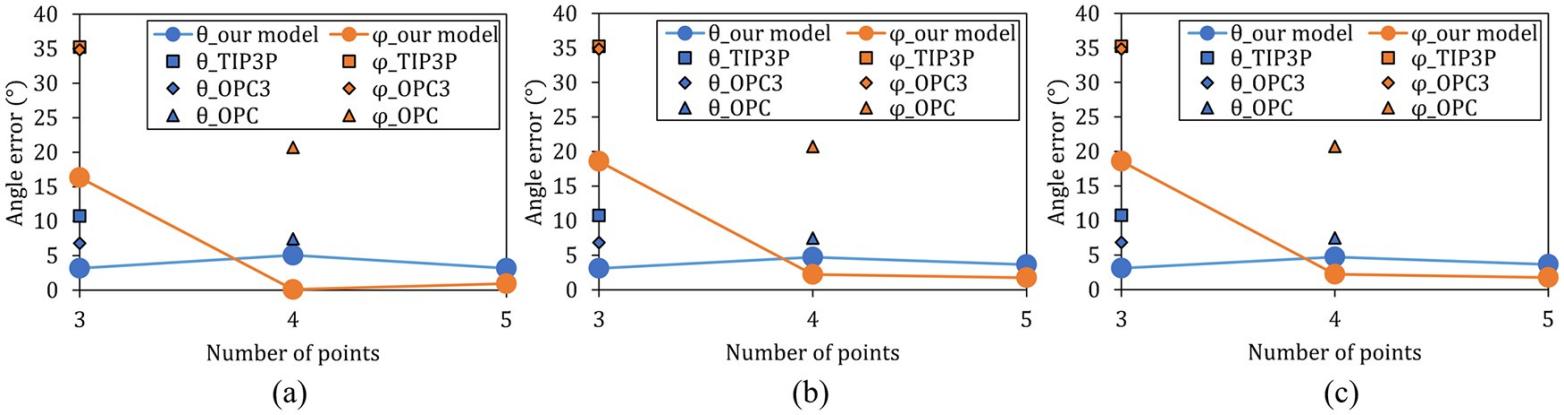
Errors in reproducing the *ab initio* water dimer geometry by the rigid water models. Our models’ *θ* and *φ* angle errors (reference values—*θ*_0_ = 57.9° and *φ*_0_ = 55.6°—are from the benchmark *ab* *initio* calculation by Klopper et al. [39]) are represented with blue and orange connected circles. In (a), (b) and (c) are the results of models built with gas exp., gas QM and liquid MP2/4MM multipole moments (Table 1) respectively. Water dimers of TIP3P, OPC3 and OPC model are energy minimized with O-O distance fixed at 2.91Å, and their angle errors in the dimers are shown as square, diamond and triangle respectively.

Accuracies of these models with respect to reproducing our main reference are compared in Fig 9. For reference, TIP3P-, OPC3- and OPC-based dimers are also tested and included in Fig 9. The water dimer geometry error values of the 4- and 5-point models are considerably smaller than that of 3-point models. The remaining errors are at ∼5° level, well below the ±10° experimental error margin of the reference values [38]. Note that this is the result of optimizing the electrostatics only. Additionally, our models have smaller errors than the existing models (TIP3P, OPC3 and OPC, Fig 9), which is not unexpected because the latter ones were optimized for liquid water instead of the water dimer. This relatively high accuracy of the “electrostatics only” optimization emphasizes the critical importance of getting right the distribution of charge in a water model. The result also suggests that one can improve a rigid water model significantly by optimizing its electrostatics, even though some physics is still missing from the model.

The dimers of 4-point water model show notably better agreement with the reference data than the dimers based on 3-point model—error of the two key angles reduced to ∼5° from ∼20°. This improvement of accuracy from 3-point to 4-point model is robust, seen for all reference sets used to build these models. However, further increasing n from 4 to 5 only has minimal effect on improving the agreement with the reference dimer geometry. The 5-point models with “gas exp.” and “gas QM” data improve from their 4-point counterparts with marginal changes (<1°). The “liquid MP2/4MM” 5-point model even has a slightly inferior accuracy compared with its 4-point model (error is 1° larger). Thus, adding more points beyond *n* = 4, which increases the accuracy of reproducing multipole moments, n-point rigid models still cannot improve much from what a 4-point model can already do. As seen from Fig 9, all of the above conclusions are independent of the specific reference multipole set used.

### Limited optimal 3-point polarizable model—only polarizability optimized

One obvious physical feature missing from rigid *n*-point water models is electronic polarizability [29, 49, 57–62]. This missing physics may be the reason why there is little, if any, improvement in the accuracy of the rigid model in reproducing water dimer as *n* is increased beyond *n* = 4, Fig 9. How much of an accuracy improvement can be achieved by adding the missing polarizability to the model? To answer this question, we compare two different approaches of improving rigid water models.

So far, in the rigid models we built, the geometry and point charge values are optimized (with OPCA method [37], by varying the monomer dipole moment of the water model) specifically for water dimer to compensate the missing gas-to-condensed-phase polarization. Now, we add polarizability to a 3-point model, whose monomer multipole moments (including the dipole) fit the reference for the monomer, then optimize the added polarizability against water dimer to the same standard—error of dimer total dipole moment smaller than 0.1%. This way, an apple-to-apple comparison is established between the two approaches to improve a rigid water model: 1) adding the missing physics of electronic polarizability; 2) compensating for its absence by optimizing the electrostatics. Either approach has only one parameter for optimization so they are competing on a level field.

**Fig 10:**
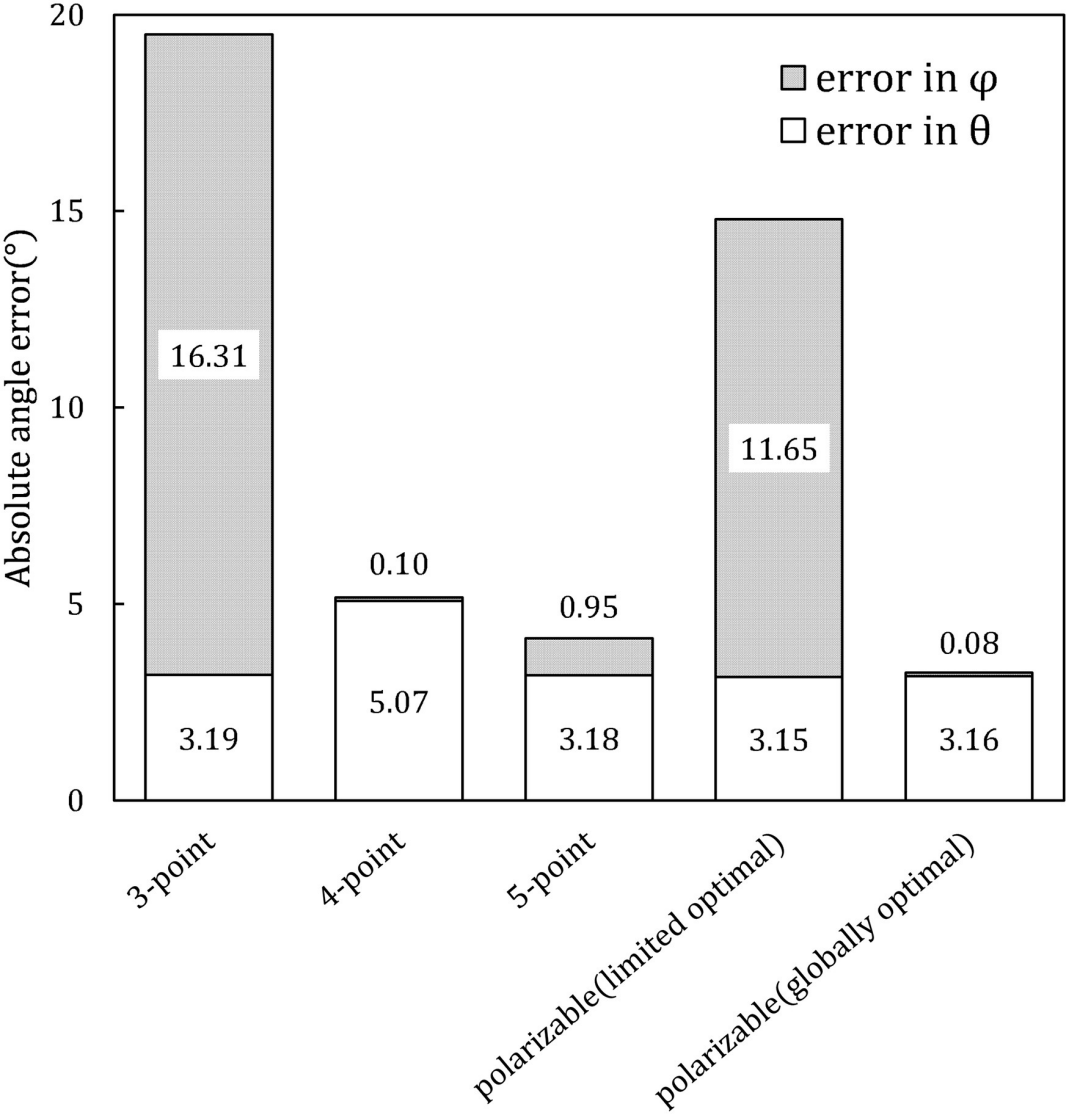
Absolute errors of the two key angles (*θ* and *φ*, Fig 2) in a water dimer made of rigid or Drude polarizable model molecules. These errors are calculated with reference to the *ab* *initio* calculation values (*θ*_0_ = 57.9°, *φ*_0_ = 55.6°) [38, 39]. Results of the 3, 4 and 5-point rigid water model dimers are built based on the “gas exp.” reference (Table 1), same with the polarizable models.

Parameters of the resulting polarizable model are shown in Table 3. The accuracy of the resulting 3-point limited optimal polarizable model is compared to that of our rigid models in Fig 10.

As illustrated in Fig 10, this limited globally optimal 3-point polarizable model has ∼5° smaller total angle error than the 3-point rigid model. This means that under the same optimization standard, adding and optimizing polarizability (while keeping the monomer dipole moment fixed to its gas phase value) is more beneficial than compensating the lack of polarization by only optimizing the dipole moment of the rigid model.

Moreover, because the monomer dipole moment is fixed to the gas phase value in this limited optimization process, the resulting polarizable model reproduces correct gas phase multipole moments when in gas phase, unlike rigid models whose dipole moment is changed for condensed phase optimization. Also, the optimized polarizability is 0.94Å^3^, 35% lower, but still qualitatively similar to the gas phase experimental value—1.44Å^3^.

However, this limited global optimal 3-point polarizable model is still inferior to the 4- and 5-point rigid models with quite a margin (∼10° higher total angle error). Therefore, adding polarizability alone cannot fix the defects of suboptimal electrostatics of a 3-point model.

With the computational cost in mind (in real simulations, 3-point polarizable model is even slower than the corresponding 4-point rigid models), this result is not very satisfactory. It suggests that the optimization protocol in which the fixed-charge base model is ignored and only polarizability is optimized has significant limitations.

### Globally optimal 3-point polarizable model—Both the polarizability and the “rigid base” are optimized

So far, we have focused on purely conceptual questions about water models’ limitations in reproducing liquid water, mimicked here by water dimer. In practical MD simulations, water models are mostly used to simulate the solvent in liquid phase. Therefore from practical perspective of water model design, we do not have to keep the rigid base of a polarizable model unoptimized. Also, the results of the limited optimal 3-point polarizable model make it clear that only optimizing polarizability is not sufficient to improve a water model’s accuracy beyond the rigid *n*-point model’s limitations. Therefore, here we build a globally optimal 3-point polarizable model, in which two parts are both optimized for the water dimer scenario: the geometry and point charge values of the rigid base (through OPCA [37], varying the monomer dipole); and the polarizability introduced by the Drude oscillator.

As discussed in “Methods” section, under the same optimization standard (reproducing dimer total dipole) a polarizable model has one more degree of freedom—the added polarizability—than the rigid models. By optimizing both its rigid base and its polarizability, we are utilizing this additional degree of freedom afforded by the polarizable model. In other words, this 3-point polarizable model is optimized with two parameters: polarizability(*α*) and gas phase monomer dipole moment(*μ*). The result is shown in Fig 10. In comparison with the 3, 4, 5-point rigid models and the limited optimal polarizable model, this globally optimal polarizable model’s result is shown along side with them as the rightmost column.

With both polarizability and gas phase monomer dipole moment optimized, the dimer geometry error of this fully optimal polarizable model shows a significant improvement from the limited optimal polarizable model, and is more accurate than the corresponding 4- and 5-point rigid models—the *φ* error is very close to zero and the *θ* error is at the same level or smaller than that of all other models. Not only the accuracy of the globally optimal polarizable model is considerably higher than that of all rigid models and the other limited optimal polarizable model, it is worth noting that this globally optimal polarizable model has a gas phase monomer dipole moment of 1.7258D, not far from the gas phase experimental value of 1.86D. The resulting polarizability is 2.29Å^3^, which is considerably larger than the experimental 1.44Å^3^. This deviation of the optimized polarizability value points to remaining deficiencies of the specific polarizable water model, likely that the model is too simplistic or/and that other physical effects not explicitly considered here (e.g. charge transfer) play a noticeable role. For the 3-point polarizable water model optimized for water dimer, decreasing the monomer dipole moment by 7% (from 1.86D to 1.7258D) resulted in a 144% polarizability increase (from 0.94Å^3^ to 2.29Å^3^). This implies that the water dimer properties are less sensitive to the model’s polarizability than to its monomer dipole moment, which agrees with the study of Soetens and Millot [63]. Although the water dimer properties are less sensitive to the water model’s polarizability, the polarizability is still very important in improving the model’s accuracy, since a 3-point polarizable model outperforms a 5-point non-polarizable model by quite a margin.

The combined results show the full potential of a polarizable water model when properly optimized. Under the same optimization standard (reproducing dimer total dipole) and with the additional degree of freedom utilized, the 3-point polarizable model outperforms 3, 4 and even 5-point rigid models by achieving the smallest error in reproducing water dimer geometry, while still having electrostatic properties in gas phase close to experimental values. At the same time, even this globally optimal polarizable model is not perfect.

## Conclusion

In order to study possible novel avenues for optimization strategies aimed at improving accuracy of explicit water models for biomolecular simulations, different “toy” water models were constructed and examined with respect to their ability to reproduce properties of water dimer—a mimic of the condensed state of water. Specifically, we constructed rigid models with 3, 4 and 5 points, and two different 3-point polarizable models with a single Drude particle to mimic electronic polarizability The models were built to match reference multipole moments, including *ab-initio*, and were then optimized to reproduce, as closely as possible, the total dipole moment of water dimer. The ability of the models to reproduce the water dimer geometry was used as the metric of the model accuracy.

First, we conclude that optimizing the “electrostatics” (charge distribution) alone can deliver high accuracy of the water model: the geometry of the resulting water dimer is essentially within 5° of the *ab* *initio* reference, this remaining error is almost half of the reported experimental error margin (±10°) on the dimer geometry. Not unexpectedly, the resulting water models show smaller errors than the existing models (TIP3P, OPC3, OPC), which were optimized for liquid water instead of the water dimer. This result reinforces the notion that optimizing electrostatics is key to water model quality, and an “electrostatically globally optimal” water model can be quite accurate.

Second, we conclude that for rigid *n*-point water models, increasing the number of interaction points from *n* = 3 to *n* = 4 can easily bring better accuracy, while by further increasing *n* from 4 to 5 only a marginal improvement can be achieved. Considering the steep increase with *n* of the computational cost associated with performing simulations based on *n*-point water model, a very convincing justification is needed for renewed efforts to build rigid water models with 5 or even more point charges. Justification for such models may need to be based on expected or demonstrated improvements in specific areas, e.g. where the possibility that non-planarity of a water models may be critical, such as phase transition between ice and liquid water that TIP5P model has a melting point much closer to experiment than most 4-point models [64]. The above conclusions are robust to the specific type—out of three different ones—of the reference multipole set used to build the models.

Next, we have investigated the effect of adding electronic polarizability to the rigid base model. Our first exercise was aimed at quantifying how much extra accuracy one can gain by having a dedicated mechanism (Drude model) to account for the polarizability. To make an apples-to-apples comparison, we compared two optimization options that employed just one adjustable parameter. The first option is the method we already applied to build our rigid models where point charge placements are adjusted to compensate for the polarizability in an average sense; the second option is to add and optimize polarizability of the Drude oscillator without changing the rigid “base” model. The second option bears similarity to optimization strategies used in practice to construct Drude polarizable models. The same reference multipole moments and dimer geometry accuracy metric were used in both options. The results show that for the same 3-point “base”, having the dedicated polarizability component does achieve a better accuracy than the purely rigid model, which is an argument in favor of polarizable models, despite their extra complexity. At the same time, the accuracy of this limited optimal 3-point polarizable model falls significantly short of the optimal 4-point rigid non-polarizable model. Apparently, the correct physics added to the rigid model via electronic polarizability is mostly “wasted” on correcting the deficiencies of the 3-point “base” model.

To reveal full potential of polarizable models, we have explored a 3-point polarizable model in which both the base charge distribution and the polarizability are globally optimized simultaneously—to the best of our knowledge, this strategy has not yet been used to build existing polarizable models used in practical simulations. The resulting truly globally optimal 3-point polarizable model easily outperforms 4- and 5-point globally optimal rigid models. Not only that the globally optimal polarizable model is more accurate in reproducing the water dimer, its unpolarized monomer dipole moment (1.7258D) is quite close to the correct gas phase value (1.86D), indicating that the model is a decent mimic for both the condensed phase and gas phase of water.

We stress that all of the water models developed in this work are not intended for use in actual atomistic simulations. Instead, these “toys” models serve only one purpose—to compare various model optimization strategies on the same footing and suggest avenues to consider for future practical optimization efforts to improve realistic water models. In the future, we plan to build and explore a truly electrostatically globally optimal polarizable model, following the over-all optimization strategy outlined in this work.

## Supporting information

S1 Protocol AMBER files for each water model we built in this study. Water dimer minimization results and input files that can be used for reproducing the results are included.
(ZIP)
